# Enhanced Immunogenicity, Mortality Protection, and Reduced Viral Brain Invasion by Alum Adjuvant with an H5N1 Split-Virion Vaccine in the Ferret

**DOI:** 10.1371/journal.pone.0020641

**Published:** 2011-06-07

**Authors:** Robert Colby Layton, Andrew Gigliotti, Penny Armijo, Leslie Myers, Jennifer Knight, Nathaniel Donart, John Pyles, Sarah Vaughan, Jennifer Plourde, Ndingsa Fomukong, Kevin S. Harrod, Peng Gao, Frederick Koster

**Affiliations:** Infectious Diseases Program, Lovelace Respiratory Research Institute, Albuquerque, New Mexico, United States of America; Centers for Disease Control and Prevention, United States of America

## Abstract

**Background:**

Pre-pandemic development of an inactivated, split-virion avian influenza vaccine is challenged by the lack of pre-existing immunity and the reduced immunogenicity of some H5 hemagglutinins compared to that of seasonal influenza vaccines. Identification of an acceptable effective adjuvant is needed to improve immunogenicity of a split-virion avian influenza vaccine.

**Methods and Findings:**

Ferrets (N = 118) were vaccinated twice with a split-virion vaccine preparation of A/Vietnam/1203/2004 or saline either 21 days apart (unadjuvanted: 1.9 µg, 7.5 µg, 30 µg, or saline), or 28 days apart (unadjuvanted: 22.5 µg, or alum-adjuvanted: 22.5 or 7.5 µg). Vaccinated animals were challenged intranasally 21 or 28 days later with 10^6^ EID_50_ of the homologous strain. Immunogenicity was measured by hemagglutination inhibition and neutralization assays. Morbidity was assessed by observed behavior, weight loss, temperature, cytopenias, histopathology, and viral load.

No serum antibodies were detected after vaccination with unadjuvanted vaccine, whereas alum-adjuvanted vaccination induced a robust antibody response. Survival after unadjuvanted dose regimens of 30 µg, 7.5 µg and 1.9 µg (21-day intervals) was 64%, 43%, and 43%, respectively, yet survivors experienced weight loss, fever and thrombocytopenia. Survival after unadjuvanted dose regimen of 22.5 µg (28-day intervals) was 0%, suggesting important differences in intervals in this model. In contrast to unadjuvanted survivors, either dose of alum-adjuvanted vaccine resulted in 93% survival with minimal morbidity and without fever or weight loss. The rarity of brain inflammation in alum-adjuvanted survivors, compared to high levels in unadjuvanted vaccine survivors, suggested that improved protection associated with the alum adjuvant was due to markedly reduced early viral invasion of the ferret brain.

**Conclusion:**

Alum adjuvant significantly improves efficacy of an H5N1 split-virion vaccine in the ferret model as measured by immunogenicity, mortality, morbidity, and brain invasion.

## Introduction

Highly pathogenic avian influenza (HPAI) virus became widely disseminated throughout Asian wild bird populations in 2004, spread to Europe and Africa, and intermittently re-emerged in domestic bird populations [1]. Bird-to-human transmission of H5N1 strains remains inefficient, with approximately 500 H5N1 infections reported to the World Health Organization. In spite of the few reported infections in recent years and the rarity of human-to-human transmission, the high mortality rate of approximately 60% among reported infections raises great concern for pandemic potential if naturally recombinant avian strains were to acquire efficient human-to-human transmission.

Vaccination is the most effective strategy to control the spread of a pandemic influenza virus. Developing a pre-pandemic vaccine for H5N1 strains presents a number of challenges [2], [3], [4], [5], [6]. First, the world population has little prior exposure to H5 strains, reducing the opportunity for cross-reactive immunity. Second, the evolution of H5N1 strains from clade 0 to clade 9 has been relatively rapid over wide geographic regions, demanding a level of heterotypic immunity from stockpiled vaccines which is not necessary for seasonal vaccines. Third, the logistics of rapidly protecting a large population requires optimal dose-sparing design, including single dose delivery with a stockpiled vaccine. Thus, immunogenicity of an H5 vaccine must be optimized to achieve reduction of both disease and transmission [7]. The vaccine composition and production would ideally be identical to that for licensed seasonal influenza vaccines by having low viral protein concentration and used without adjuvant.

Low doses of unadjuvanted, inactivated whole virus H5N1 vaccines appear to be immunogenic in humans and protective in immunized ferrets [8], [9]. Development of split-virion preparations for seasonal vaccine is now preferred over the whole virion formulations due to reduced reactogenicity yet retained immunogenicity among children [4]. There is, however, mounting evidence that the immunogenicity of split-virion H5 vaccines may be inferior to whole virion vaccine formulations. Two doses of unadjuvanted split-viron H5N1 vaccine failed to induce homologous antibody and failed to protect ferrets against heterologous challenge [10]. Likewise two immunizations of an unadjuvanted split-virion vaccine (7.5 µg and 15 µg doses) from A/Vietnam/1194/2004 (H5N1) strain failed to stimulate antibody or reduce fever and viral shedding and provided only 50% protection against lethal challenge [11]. In macaques, unadjuvanted split-virion formulation failed to stimulate serum antibody [12]. In children and adults, an unadjuvanted split virion H5N1 vaccine formulation induced protective levels of antibody in only half of the subjects [13], [14].

Improvement in immunogenicity has been attained by additional booster doses [15], higher initial priming doses [13], or administration with an immunological adjuvant. Several oil-in-water based adjuvants have been tested for safety and efficacy in humans [5]. Other vaccine strains have been previously shown to be protective in the ferret model when administered in the presence of other adjuvants [11], [16]. Addition of an oil-in-water adjuvant to a split-virion H5N1 vaccine in ferrets reduced morbidity and viral shedding and completely protected against mortality after a lethal heterologous strain challenge [11]. However, alum, an aluminum salt compound, is the only adjuvant to be used without the addition of other components with a vaccine licensed in the United States until 2009. Compared with unadjuvanted vaccine, split-virion H5N1 vaccine administered with alum to children resulted in higher titers of hemagglutination inhibition antibody [14], [17].

We used the ferret model to test whether the split-virion H5N1 Clade 1 vaccine formulation induced protective immunity without and with an alum adjuvant.

## Materials and Methods

### Ethics Statement

All procedures were conducted under protocols approved by the Institutional Animal Care and Use Committee (IACUC) at Lovelace Respiratory Research Institute, and all facilities were accredited by the Association for Assessment and Accreditation of Laboratory Animal Care International (AAALAC). To ameliorate suffering, animals that were not expected to survive until the next observation period moribund as described in the observation section) were humanely euthanized.

### Ferrets

Castrated ferrets (*Mustela putorious furo*, Triple F Farms, Sanger, PA) aged 6–8 weeks were held for fourteen days for acclimation and quarantine. Animals were pair- or triple-housed in plastic bottom rabbit/ferret cages (Allentown Inc., Allentown, NJ) during quarantine and vaccination period in the ABSL2 area and in stainless steel cages (Allentown Inc, Allentown, NJ) modified to have LEXAN® doors to prevent intercage contact after viral challenge. During the live virus exposure period, housing and manipulation of animals was performed within a bioBubble® (bioBubble Inc, Fort Collins, CO) inside an ABSL3+ containment area. Animals were screened for antibody to currently circulating influenza A and B viruses prior to shipment from the supplier and prior to their move into the ABSL3+ Facility, and only seronegative animals were used. A total of 112 ferrets were randomized (PathTox 4.2.2, Xybion, Cedar Knolls, NJ) into groups of fourteen animals per vaccination group and divided between two vaccine trials, hereafter designated as the ‘unadjuvanted trial’ and the ‘adjuvanted trial’ (**Table 1**). Each vaccine trial was divided into two cohorts with seven animals per vaccination group during each of two exposure experiments. Six additional animals from the alum-adjuvanted vaccine trial were challenged six months after vaccination. An additional 40 ferrets were vaccinated, challenged, and euthanized 2 days or 6 days after challenge for the purpose of RNA transcriptional microarray analysis; those results will be reported elsewhere.

**Table 1:** Summary of vaccine trial group allocations.

Trial^a^	Group	Vaccine Dose (µg)	Alum	N	Challenge Interval^b^
U	1	30	No	14	3
U	2	7.5	No	14	3
U	3	1.9	No	14	3
U	4	Saline	No	14	3
A	1	22.5	Yes	14	4
A	2	7.5	Yes	14	4
A	3	22.5	No	14	4
A	4	Saline	No	14	4
A	5	22.5	Yes	2	24
A	6	7.5	Yes	2	24
A	7	22.5	No	2	24

a “U” were animals in the Unadjuvanted Vaccine Trial. “A” were animals in the Adjuvanted Vaccine Trial.

b Challenge interval is from the second vaccination until intranasal instillation of virus.

### Vaccines

H5N1 Monovalent Influenza Subvirion Vaccine (rgA/Vietnam/1203/2004) was provided by DMID/NIAID/NIH. Vaccines were provided at three concentrations of hemagglutinin protein, specifically 90 µg/mL unadjuvanted (lot U10915C), 90 µg/mL unadjuvanted (lot UD07827), 90 µg/mL alum-adjuvanted (lot UD07828), and 30 µg/mL alum-adjuvanted (lot UD07826).

Vaccinations for the unadjuvanted trial used the 90 µg/mL unadjuvanted (lot U10915C) diluted in physiological saline immediately prior to vaccination to 30 µg, 7.5 µg, and 1.9 µg, within 0.5 mL. The adjuvanted trial used 0.25 mL of the vaccine as received to immunize the animals with 22.5 µg with (lot UD07827) and without (lot U10915C) adjuvant, and 7.5 µg with adjuvant. Control animals received either 0.5 mL or 0.25 mL of physiological saline. Vaccinations were delivered by intramuscular injection of the thigh with the other leg used for the second vaccination.

Six or eight weeks (unadjuvanted or adjuvanted trial, respectively) prior to intranasal challenge blood samples were collected and animals received the first of two vaccinations. Ferrets were vaccinated again three or four weeks later (unadjuvanted or adjuvanted trial, respectively). Two vaccination schedules were used to provide an additional test of the vaccine formulations during the adjuvanted trial (the efficacy of vaccine with increased time from vaccination to challenge). One week prior to challenge blood was drawn for antibody titers. Five days prior to challenge, animals were moved into ABSL3+ containment for acclimation and pre-instillation observations and data collection.

### Virus

Influenza virus A/Vietnam/1203/2004 (H5N1) (VN/1203) was obtained as a low-passage stock from the Centers for Disease Control and Prevention. This stock was passaged once in 10-day embryonated chicken eggs to generate the master stock and once again in eggs to generate the virus for all subsequent challenges (Lot 03142007EP2.2). Aliquots of 0.5 mL to 1.0 mL were stored at −80°C. After storage, the virus was determined to have a concentration of 1.4×10^8^ plaque forming units (PFU)/mL, 5.8×10^8^ 50% tissue culture infectious dose (TCID_50_)/mL, and 1×10^8^ 50% egg infectious dose (EID_50_)/mL. Influenza A/Vietnam/1203/2004 is a Risk Group 3 pathogen; all manipulations were carried out in a BSL3/ABSL3+ containment facility.

### Intranasal Challenge

Ferrets were anesthetized by intramuscular injection with a mixture of ketamine (Ketaset®, Fort Dodge Animal Heath, Fort Dodge, IA) and xylazine (AnaSed®, Lloyd, Shenandoah, IA) to give 20 mg ketamine/kg and 4 mg xylazine/kg. Sedated animals were tested for a sneeze reflex using the filled instillation syringe and 20 ga catheter (Surflo®, Turmo Medical Co. Elkton, MD) prior to drop wise instillation. Animals were held by the scruff with the nares up and tilted slightly so as to have the inoculum flow along the nasal turbinates. A total of one milliliter (500 µL per nares), was used to challenge the animals with 10^6^ PFU for the first cohort of the first vaccine trial or 10^6^ EID_50_ for the second cohort of the first trial and both cohorts of the second trial. Back-titration was performed on challenge stock and diluted instilled virus to ensure consistent instillations between the five exposure cohorts.

### Observations

Twice daily observations to assess animal health by determining ocular discharge, nasal discharge, sneezing, coughing, stool characteristics, and activity score. An activity score (0 = alert and playful, 1 = alert but playful only when stimulated, 2 = alert but not playful when stimulated, and 3 = neither alert nor playful when stimulated) was obtained each time the ferrets were observed. Daily weights and temperatures were also collected. Temperatures were obtained from shoulder and hip implanted microchips (IPTT-300 Implantable Programmable Temperature and Identification Transponder; Bio Medic Data Systems, Inc, (BMDS) Seaford, DE). Moribund animals were designated by any one of the criteria: a temperature of less than 33.3°C, weight loss equal to or greater than 25%, unresponsiveness to touch, self-mutilation, paralysis, movement disorder, or respiratory distress.

### Viral Load Samples

Nasal wash was performed by using a total volume of one milliliter (0.5 mL/nares) of phosphate buffered saline (PBS) containing 1% bovine serum albumin, 100 units penicillin/mL, 100 µg/mL streptomycin, and 0.25 µg amphotericin B/mL. Samples werecollected prior to first vaccination, one week prior to challenge, on days one through four post-instillation (pi), day six, and prior to moribund or scheduled euthanasia.

Necropsy was performed to obtain tissue samples for virological and histological examination on animals found dead, moribund, or at 14 pi. Tissues were collected according to a standardized protocol not based on gross pathology.

Tissues for viral load were a lavage of a lung lobe, lung (four 250 mg sections, a hilar and peripheral section each from a caudal and a cranial lobe), 250 mg brain, 250 mg spleen, complete tracheal bronchial lymph nodes, and two tracheal rings. Tissues were placed into sterile PBS with one 5 mm stainless steel bead (two beads for lung) and homogenized for four minutes using the TissueLyser (QIAGEN, Valencia, CA). An aliquot of homogenate was then used to isolate RNA for viral load quantification by qRT-PCR. Remaining homogenate was used to determine viral load by plaque.

### Viral Load by Plaque Assay

Madin-Darby Canine Kidney (MDCK) cells were used to quantify virus from samples by modified standard techniques [18]. Briefly, 6-well tissue culture plates were allowed to absorb 200 µL of 10-fold diluted virus. After removal of virus, agarose overlay was added and plates were incubated at 37°C for two days. Plaques were read after fixation, removal of the agar and staining with 1.6% w/v crystal violet. Uninfected well of each plate was used as a comparison for infected cells.

### Viral Load by RT-qPCR

Samples were processed by the Kingfisher (Thermo Scientific). BCP (1-Bromo-3-chloropropane) was pipetted into the sample (200 µl into 1000 µl) vortexed for 10 seconds, and then centrifuged at 13,000 rpm at 4°C for 10 minutes. The aqueous layer was removed, placed in sampling tube and extracted by the “Magmax Clear” protocol according to manufacturer's instructions.

Reverse transcription (RT) and quantitative PCR (qPCR) were carried out in the same reaction. Samples were run using either the ABI TaqMan One-Step RT-PCR Master Mix Reagents Kit (Foster City, CA) or the Quantitect Virus Kit from Qiagen (Valencia, CA). Samples prepared using the ABI kit were run in triplicate on a 384 well plate. Each reaction contained 2.5–5 µl of RNA sample or no template control (NTC) (3 µl of RNA or NTC and a total of 15 µl volume was found to be optimal), 33.3 nM primer and 13.3 nM probe. Samples prepared using the Qiagen kit were run using the same reaction volume as for the ABI kit, but with and 333 nM primer and 80 nM probe. A standard curve was run with an influenza M1 gene RNA in 10-fold increments ranging from 10^9^ to 10^1^ copies/µl. The standard was prepared using the blunt-TOPO vector from Invitrogen (Carlsbad, CA) and the Mega-short-Script from Ambion (Austin, TX). Reactions were set up using the Eppendorf epMotion 5075 robot. The sequence for the forward primer is TTC ACA GCA TCG GTC TCA CAG ACA, the reverse is TCC AGC CAT CTG TTC CAT AGC CTT and the probe is/56-FAM/AAC AGA ATG GTG CTG GCT AGC ACT/3BHQ_2/. Primer sequences were designed and synthesized by Integrated DNA Technologies (IDT) (Coralville, IA). RT was carried out for 20′ at 50°C. PCR conditions were 95°C for 5′, 40 cycles of 95°C for 15″, 60°C for 45″ on an ABI 7900HT Fast Real-Time PCR System machine. Data was analyzed using ABI SDS 2.3 software. After manually setting the threshold for the midpoint for each standard curve, the mean slope was −3.5 for a mean of 93.07% efficiency, the mean y-intercept was 41.5, and the mean r^2^ was 0.998 for 18 plates.

### Serology

Serological assays were modified from previously described procedures [19], [20]. Serum samples were inactivated by receptor-destroying enzyme (Enka-Seiken, Tokyo, Japan) followed by heat inactivation at 56°C for 30 minutes. Hemagglutination inhibition (HI) was performed using horse red blood cells [21]. Titers of neutralizing antibodies by the microneutralization assay (MN) were determined by inoculating MDCK cells in 96-well plates with serum that was incubated for one hour with 2000 TCID_50_/mL A/Vietnam/1203/2004 combined with 2-fold diluted serum. Geometric mean titers are reported, negative titer was denoted as 10.

### Histopathology

Lung tissue and brain with olfactory bulbs were fixed in buffered formalin. Sections were trimmed for histopathology from the left cranial, right middle and right caudal lung lobes. Sampling of brains included olfactory bulbs and coronal sections through the entire brain at the frontal region, parietal region and cerebellum with brainstem. Fixed tissues were paraffin embedded; 4 µm sections were cut, stained with hematoxylin & eosin, and read by a board certified veterinary pathologist (APG) without knowledge of the vaccination history. Standard, subjective grading of lesions inflammation was based on the severity of the change within affected areas and the extent of tissue affected by the change. Scores were:

0 (none) = essentially no tissue affected;

1 (minimal) = ∼1 to 5% affected,

2 (mild) = ∼6 to 25% affected,

3 (moderate) = ∼26 to 50% affected, and

4 (marked) = ∼50 to ∼100% affected with a severe change.

### Statistics

Statistical analyses were performed using GraphPad Prism (version 5.03, GraphPad Software, Inc. La Jolla, CA). Serum antibody response was analyzed by analysis of variance (ANOVA) using the Bonferroni post-test. Survival proportions were tested using the Log-Rank test. Morbidity by increasing activity score was examined by using Fisher's exact test. Viral load was determined to be different by the repeated measure ANOVA. Hematological measurements were analyzed by the *t*-test.

## Results

### Serum Antibody Response

**Figure 1:**
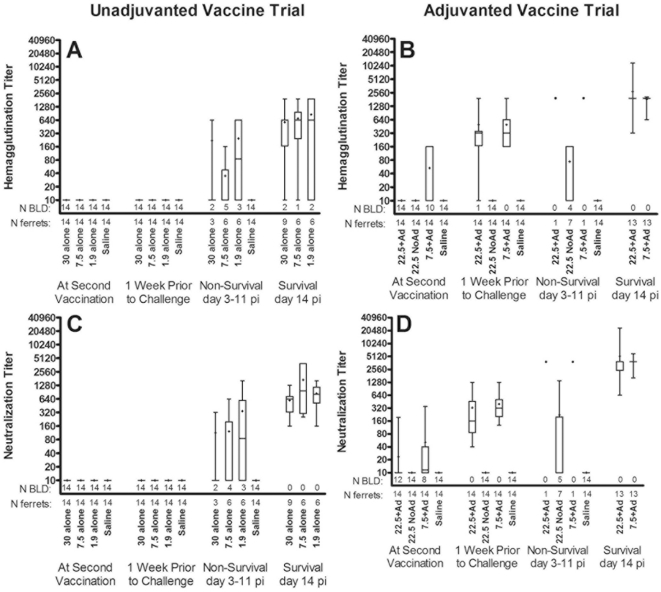
Hemaggutination inhibition and neutralizing antibody titers. Box plots denote the median (line in box), the 25^th^ and 75^th^ quartile (ends of box), the mean (+), and the 95% confidence intervals (whiskers) with dots representing individual data points. The number (N) of samples analyzed are shown below the x-axis under the number of samples that were below the limit of detection (BLD). At second vaccination is 3 or 4 weeks after initial vaccination and 1 week prior to challenge is either 2 or 3 weeks after second vaccination.

Unvaccinated controls never had detectable neutralizing antibody (**Figure 1**). Ferrets vaccinated without adjuvant, regardless of vaccine dose, did not have detectable antibody prior to challenge. Survivors vaccinated without adjuvant developed serum neutralizing antibody, with titers after challenge ranging from 160 to 3840 at day 14.

In contrast to unadjuvanted vaccine recipients, both adjuvanted vaccines elicited antibody in all recipients prior to challenge (**Figure 1 B, D**). Peak mean neutralizing antibody one week after booster immunization was greater than 512, with only a modest decrease during the 4 week interval prior to challenge. The two non-surviving recipients of alum-adjuvanted vaccine had serum titers within the same range as surviving ferrets. Neutralization titers of survivors in the unadjuvanted dose groups were significantly lower from each of the survivors in the alum-adjuvanted vaccine groups fourteen days after challenge (ANOVA with Bonferroni post-test, P<0.001).

### Survival

**Figure 2:**
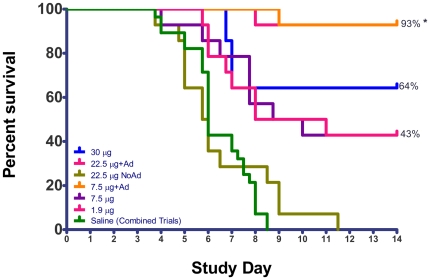
Survival proportions demonstrate adjuvant advantage. Saline vaccinated, control animals, from both vaccine trials are combined. Asterisks (*) denotes a significant difference between survival rates of 93% (adjuvanted vaccine) and all others (Log-Rank test, p<0.001).

All unvaccinated ferrets died by between day 3 and 9 pi. Recipients of the unadjuvanted 30 µg dose of vaccine had a 64% survival rate, while recipients of 7.5 µg and 1.9 µg doses had 43% survival (**Figure 2**). Survival of the unadjuvanted 22.5 µg dose group in the second trial was 0%, markedly lower than the similar dose (30 µg) in the first trial. In contrast to unadjuvanted vaccine, ferrets vaccinated with adjuvant had significantly higher survival of 93% in both groups (Log-Rank test, p<0.001).

### Morbidity after viral challenge

**Figure 3:**
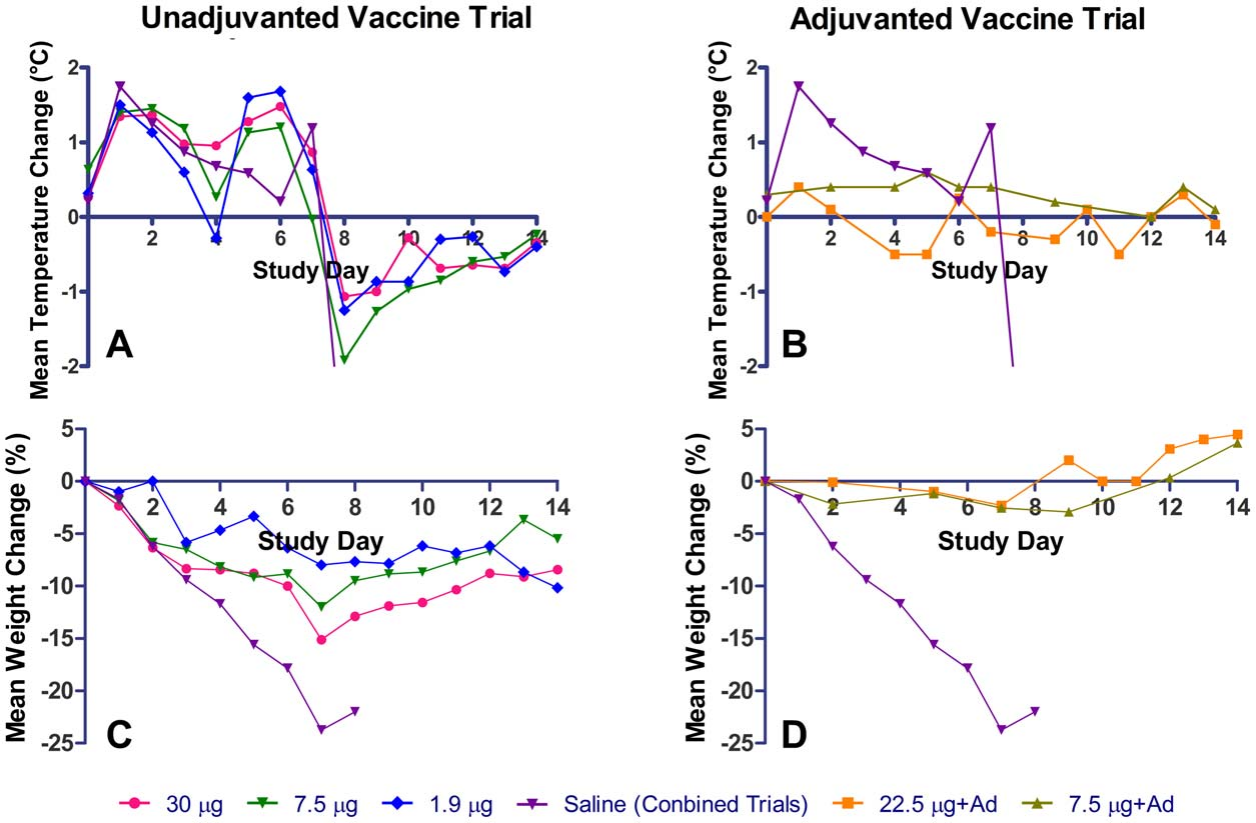
Group mean temperature and weight change of vaccinated survivors. Data represent the mean of individual animal temperature and weight change for each vaccinated group. The mean changes of the saline groups are combined from both cohorts to provide the same baseline for both trials.

Fever (>1°C above baseline) was documented in all ferrets of unadjuvanted vaccine or saline groups beginning on day 1 pi and was equivalent in duration (approximately 6 days) and amplitude among all unadjvuanted groups (**Figure 3, A & B**). In contrast, ferrets dosed with alum-adjuvanted vaccine did not develop fever at any time after challenge.

Survivors in each of the three unadjuvanted vaccinated groups lost less weight than non-survivors (**Figure 3, C**) with no significant difference between the groups (p>0.05 ANOVA for Repeated Measures). In contrast, ferrets vaccinated with adjuvant did not show weight loss post-challenge (**Figure 3, D**).

**Figure 4:**
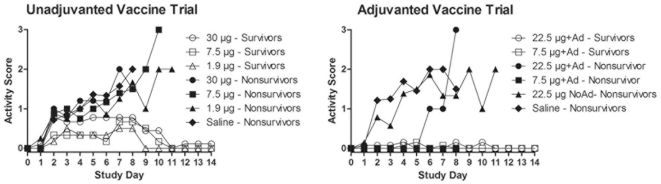
Group mean activity scores of vaccine recipients. Higher activity score indicates greater morbidity.

Thirty-five of the 42 vaccinated ferrets in the unadjuvanted vaccine trial experienced decreased appetite and liquid stool by day 2 pi compared to 1 of 28 vaccinated with adjuvant in the adjuvanted vaccine trial. Of the 56 ferrets vaccinated without adjuvant including the saline controls, 24 displayed sneezing on one or more days, typically beginning on day 3 pi compared to only 4 of 28 vaccinated with adjuvant. By day 7 pi a decrease in activity (score ≥1, **Figure 4**) was seen in 89%, 100%, and 83% of survivors (30 µg, 7.5 µg, and 1.9 µg doses, respectively) in the unadjuvanted vaccine trial. Non-survivors had further deterioration in activity one to two days prior to euthanasia for moribund condition. Survivors in either vaccine trial did not experience a decrease in activity beyond the score of 2. In contrast, only 46% and 15% (22.5 µg with adjuvant and 7.5 µg with adjuvant, respectively) of the surviving animals in the adjuvanted vaccine trial displayed any changes in behavior (activity score ≥1). Adjuvanted 7.5 µg dose group was significantly different, lower, (Fisher's exact test, p<0.01) than each of the surviving unadjuvanted dose groups.

### Hematology

**Figure 5:**
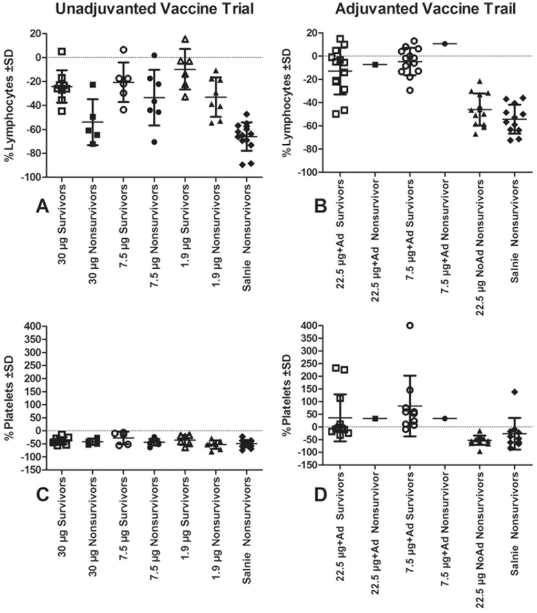
Post-instillation lymphopenia and thrombocytopenia in vaccinated ferrets. Data represent the percentage change from pre-instillation collection and samples available for analysis. Mean change and standard deviation (SD) are represented for each group.

By day 4 pi all ferrets in the unadjuvanted vaccine trial showed a significant decrease in total white blood cell count (data not shown), decrease in percentage of lymphocytes, and decrease in the platelet count (t-test significant for each comparison at p<0.05) (**Figure 5, A &B**). In contrast, the alum-adjuvanted vaccine recipients did not have thrombocytopenia or lymphopenia (**Figure 5, C & D**).

### Viral load

**Figure 6:**
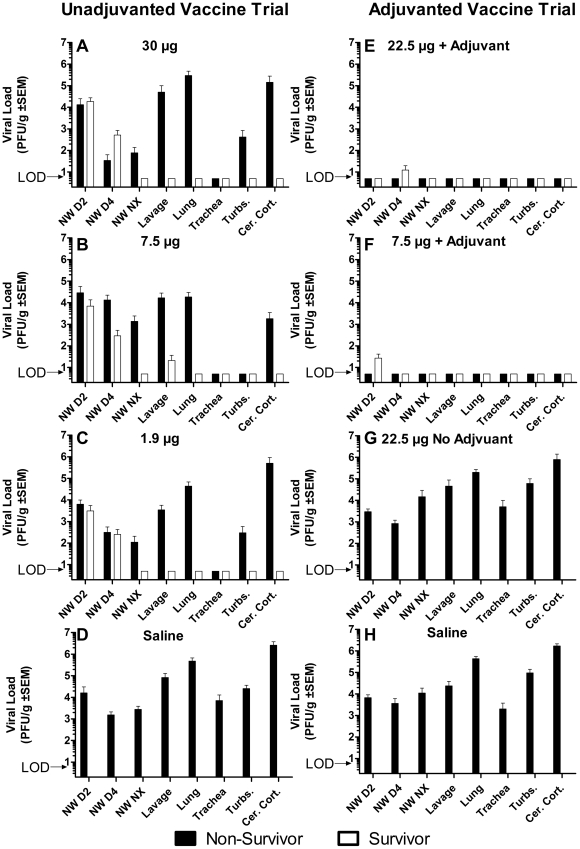
Viral load by culture in nasal wash and tissue samples. Bars without error represent samples that were analyzed but where no viral culture was found, below limit of detection (LOD). Nasal wash (NW) samples were obtained on day 2, 4, and at necropsy (Nx). Tissue samples obtained at necropsy were lung lavage (lavage), four sections of lung (lung), tracheal rings (trachea), turbinates (turbs.), and brain (cer. cort.).

**Figure 7:**
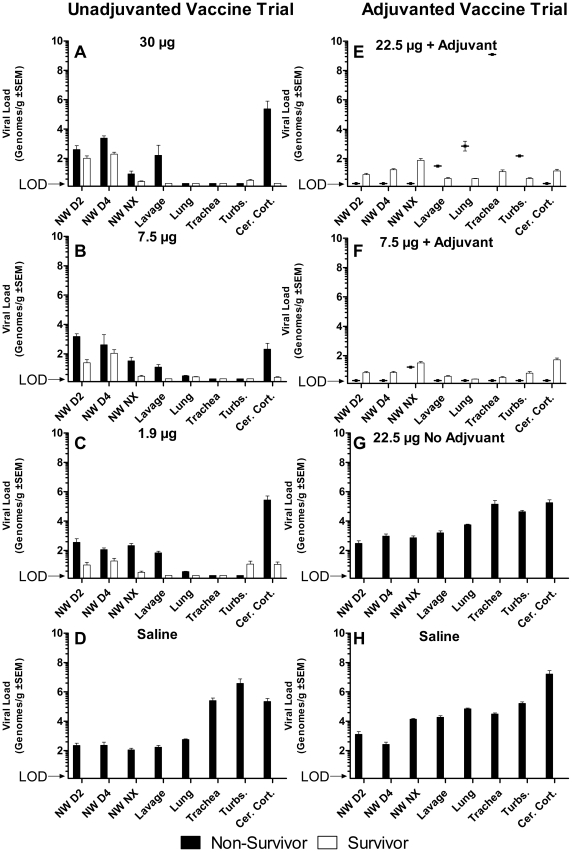
Viral load by qRT-PCR nasal wash and tissue samples. Bars without error represent samples that were analyzed but where no viral culture was found, below limit of detection (LOD). Nasal wash (NW) samples were obtained on day 2, 4, and at necropsy (Nx). Tissue samples obtained at necropsy were lung lavage (lavage), four sections of lung (lung), tracheal rings (trachea), turbinates (turbs.), and brain (cer. cort.).

Viral loads in unvaccinated control ferrets in both trials, and in ferrets vaccinated with unadjuvanted 22.5 µg dose vaccine, was high in all tissues and nasal washes when measured both by viral culture (**Figure 6; D, G, H**) and RT-qPCR (**Figure 7; D, G, H**). For all tissues there was no significant difference in titers between the unadjuvanted vaccine group and the two unvaccinated control groups (p>0.5 for each comparison, ANOVA of repeated measures). Groups vaccinated with 30 µg, 7.5 µg, and 1.9 µg of unadjuvanted vaccine also had similar viral loads (**Figure 6; A, B, C**) and RT-qPCR (**Figure 7; A, B, C**). Among non-survivors of unadjuvanted vaccine (total N = 21), there were no significant differences in viral loads in the nasal washes and necropsy tissues, with the exception of the turbinate cultures. Survivors had viral loads in nasal washes at day 2 and 4 pi not significantly different from nonsurvivors and the unvaccinated controls, indicating that intensity of upper tract infection did not predict survival. Survivors in the unadjuvanted 30 µg group did not have virus detected by culture or RT-qPCR in tissues collected at necropsy on day 14 pi. In contrast, survivors in the unadjuvanted 7.5 µg and 1.9 µg groups did have virus detected by RT-qPCR in culture negative tissues.

In the alum-adjuvanted vaccine groups, nasal washes had low viral loads on day 2 and 4 pi (**Figure 6** **&** **7; E & F**), but culture of all tissues of both survivors and nonsurvivors were negative, suggesting that the addition of alum adjuvant was associated with early suppression of upper tract infection. The one non-survivor in the alum-adjuvanted 22.5 µg dose group had viral RNA detected in respiratory tissues. However, all lavage and tissue cultures were negative, suggesting a cause of death other than aggressive avian influenza replication.

Brain tissue from non-survivors in unvaccinated and un-adjuvanted vaccine groups contained high levels of virus by culture and RT-qPCR. In contrast, all survivors had negative cultures of brain tissue. Yet, 35% of the adjuvanted groups had low levels of viral RNA in the brain. These viral load and clinical observations are consistent with a major role of replicating virus determining a fatal outcome.

### Histopathology

Lungs of non-survivor animals revealed lesions characteristic of avian influenza in the ferret, including atypical fibrinonecrotic, neutrophilic pneumonia with hemorrhage often with intervening regions of normal-appearing lung (**Figure S1**). Pulmonary lesions tended to be focused in centriacinar regions, although coalescence of pulmonary lesions to involve most of a lung lobe was common in severely affected animals. Areas of inflammation were characterized by focally extensive necrosis of parenchyma with fibrin exudation, neutrophil and macrophage influx and hemorrhage.

While all regions of the brain examined were typically involved to some degree, the olfactory bulb and the parietal lobe were always affected in all non-survivors (**Figure S2**). Brains showed lesions typical of avian influenza in this model, including lymphohistiocytic and necrotizing meningoencephalitis, multifocal in distribution with relatively discrete lesioned areas. Areas of inflammation tended be intensely necrotizing with abundant pyknosis and karyorrhexis of cells and increased cellularity with lymphocytes, histiocytes and neutrophils. The meningeal component of inflammation was often less than the encephalitis. Lesions were often worse in olfactory bulbs and more rostral and ventral brain sections with decreasing severity in caudal sections.

**Figure 8:**
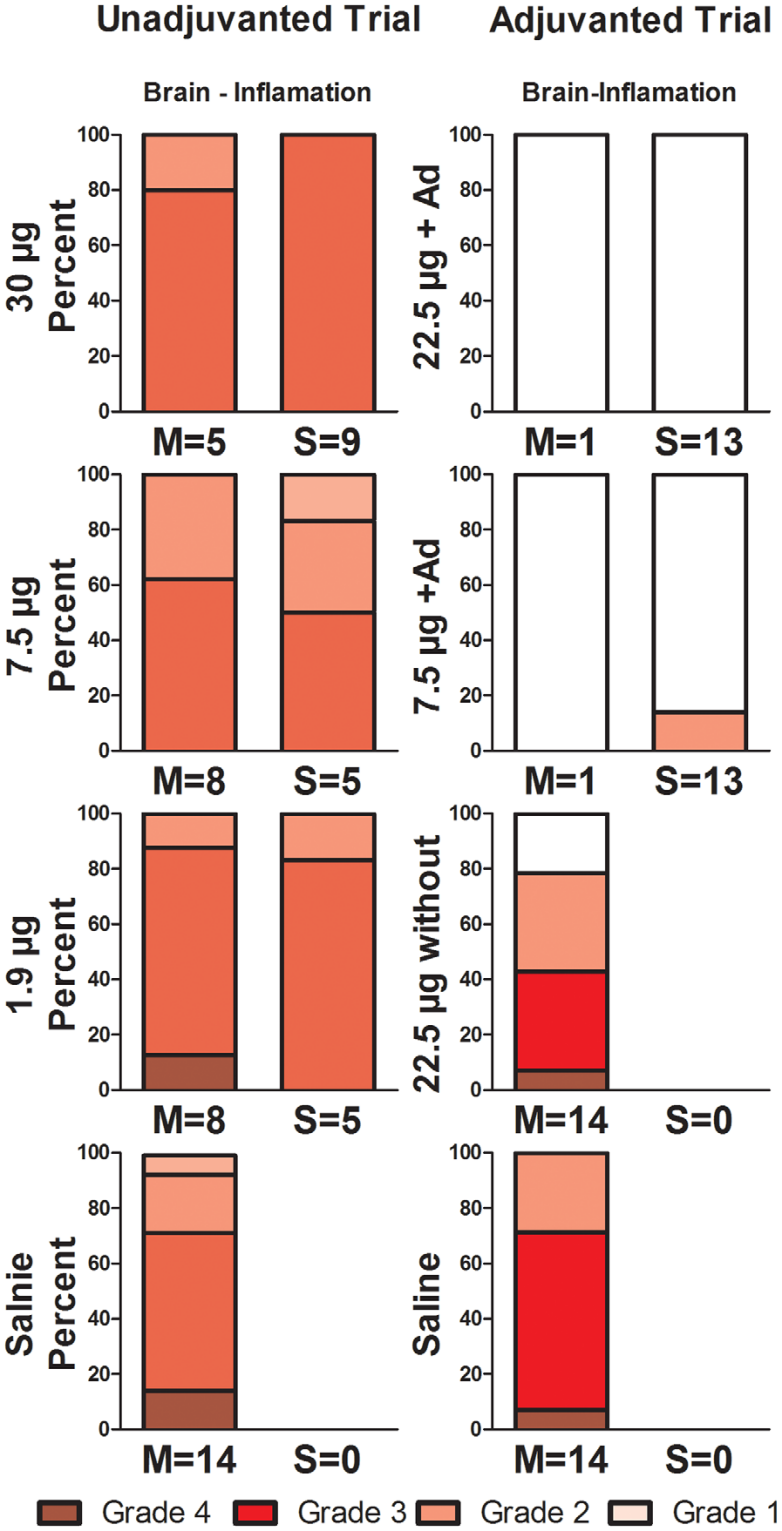
Histopathology grading of brain lesions in survivors and non survivors. “Brain-Inflamation” represents sections of either olfactory bulb or cerebral tissue with evidence of active and/or resolving inflammation. Tissues from moribund animals are denoted with an “M”, those from survivors (day 14 pi) are denoted with an “S”; the number after the “M” or “S” indicated the number of ferrets in that group. The Y-axis is the percent within each group (M or S) that was scored to each inflammation severity/grade. Grade 4 represents the most inflammation, whereas Grade 1 represents the least inflammation. Grade 0 is clear and represents that no inflammation was found.

Most of the survivors in both the unadjuvanted trial (15 of 19) and the adjuvanted trial (24 of 26) had some degree of resolving pneumonia at day 14 pi, indicating that challenged animals developed pneumonia regardless of vaccination status (**Figure S3**). The striking comparison was found in brain tissue (**Figure 8**). Moderate brain inflammation and/or malacia (necrosis with loss of brain tissue) was seen in all 19 survivors of the unadjuvanted vaccine groups, despite the negative cultures (**Figure 6**). In marked contrast only 2 of the 26 survivors in the alum-adjuvanted vaccine groups had evidence of brain inflammation (p<0.001 by Mann-Whitney rank test). The histopathologic findings of no or minimal inflammation in olfactory bulb and brain of adjuvanted trial survivors is consistent with either prevention of brain invasion or subsequent viral replication within the brain.

### Viral challenge six months after vaccination

Six months after vaccination with alum-adjuvanted vaccine, neutralizing antibody was below detection limits in the four ferrets tested (**Table 2**). After challenge, all 4 alum-adjuvanted vaccine recipients survived to day 14 pi, whereas both recipients of unadjuvanted vaccine were moribund by day 7 pi. All 6 animals became febrile after viral challenge, although the alum-adjuvanted vaccine ferrets developed fever on day six, compared to unvaccinated animals developing fever by day two. Significant weight loss of 24% occurred in one alum-adjuvanted vaccine recipient whereas the other three recipients did not lose weight. Virus was cultured from throat swabs from vaccine recipients of unadjuvanted (days 1–4 and 6 pi) and 22.5 µg alum-adjuvanted (days 1 and 3 pi). Although the numbers of ferrets studied were small, the data suggest somewhat higher levels of morbidity compared to ferrets challenged one month after the alum-adjuvanted booster dose.

**Table 2:** Summary of results from challenge six months after vaccination.

Vaccination	Titer w-21^a^	Titer w-1^b^	>10% weight loss	Fever	Throat swab	Survival
22.5 µg+Ad	1280, 806	<20	0 of 2	Yes	2 of 2	2 of 2
7.5 µg+Ad	<20, 508	<20	1 of 2	Yes	0 of 2	2 of 2
22.5 µg NoAd	<20, <20	<20	2 of 2	Yes	2 of 2	0 of 2

a w-21 is four weeks after second vaccination.

b w-1 is one week prior to intranasal challenge.

## Discussion

We show here in the ferret model that a split-virion H5N1 vaccine elicited poor protective efficacy in the absence of adjuvant (**Figure 2**). Inclusion of alum adjuvant not only protected ferrets against a homologous lethal challenge, but also prevented most measures of morbidity and histopathologic evidence of brain invasion in surviving animals (**Figure 8** **, S2**). The relevance of these findings to estimating avian influenza vaccine efficacy in humans depends on three features of the test system: the ferret as a test model, the immunogenicity of the H5 hemagglutinin in the human compared to the ferret, and the effect of the adjuvant on improving immunogenicity in the ferrets compared to the human.

The ferret model is considered to be the gold standard to test vaccine efficacy, since the ferret disease mimics human disease with respect to inhalation transmission, incubation period, tissues infected, and histopathologic response to the influenza virus [22], [23], [24], [25]. The ferret has a density and array of sialic acid receptors similar to humans [26], [27], [28], and therefore expresses similar susceptibilities to infection by H5N1 strains [26], [29]. Only a few strains of H5N1 HPAI have been shown to be fatal in ferrets [30]. The strain we used, A/Vietnam/1203/2004, has been shown to be fatal in ferrets in intranasal doses from 10^1^ to 10^7^ EID_50_ [16], [30], [31], [32]. The high level of virulence of the VN/1203 strain in ferrets [29] is attributable to multiple viral elements including the multibasic amino acids in the HA hinge region and replication competence expressed in the polymerase enzyme complex [33].

The ferret serum antibody response to vaccination is similar to that in vaccinated humans [24]. Modest immunogenicity inherent in H5 hemagglutinins was seen in the initial immunogenicity trial of the unadjuvanted VN/1203 split virus vaccine [13]. The highest 2-dose regimen elicited an MN GMT >1∶40 in only 54% of healthy adults, and the standard prime-boost dose with 15 ug elicited MN titers at this level in only 22% of adults. Even after a third dose of 15 ug, only 43% of vaccinees had an MN titer >1∶40, and only 27% had an HAI GMT titer >1∶40 [15]. In an early test of immunogenicity with a split-virion vaccine closely related to the clade 0 Hong Kong/97 viruses, two doses of 30 µg of unadjuvanted hemagglutinin protein seroconverted only a few young adult volunteers [34]. A split-virion vaccine based on the H5N1 clade 1 strain A/Vietnam/1194 was also poorly immunogenic without adjuvant [35].

Vaccination of ferrets with unadjuvanted split-virion vaccine resulted in no serum antibody to H5 by the hemagglutination-inhibition and microneutralization assays (**Figure 1**). Other investigators have documented the failure to stimulate serum antibody in vaccinated ferrets [2], [10], [11]. Immunization with two 7 µg doses of HA from an avian H5N3 strain failed to induce antibodies to VN/1203 [36].

A wide array of experimental vaccines and adjuvants for prepandemic and seasonal use have been shown to be immunogenic [37], but use in the near future will focus on proven technologies. Aluminum salts are the only immunological adjuvants approved in the United States [38]. Most immunogenicity studies in humans have been done with aluminum hydroxide (aluminum oxyhydroxide) although aluminum phosphate and alum (containing aluminum potassium disulfate) have also been used. The adjuvant activity of aluminum salts is mediated as an antigen depot, induction of local inflammation attracting antigen-processing cells to the site, and activation of the Nalp3 inflammasome. Adjuvants likely mediate their effects through a wide array of immune networks [39] although aluminum salts have not yet been studied by network analysis.

In ferrets the addition of alum adjuvant has increased the immunogenicity of the split virion vaccines. A single 7 µg dose of whole virus H5N1 vaccine with alum adjuvant stimulated high titers of neutralizing antibody and protected ferrets from lethal challenge [16]. Likewise a single 15 µg dose of split virus vaccine with aluminum phosphate adjuvant stimulated antibody and completely protected ferrets [11]. Novel oil-based and polymer-based adjuvants combined with single low-dose immunizations have also been shown to stimulate antibody and provide protection from lethal challenge [10], [11], [24]. Macaques immunized with an alum-adjuvanted split-virus H5N1 inactivated vaccine developed functional antibody titers and were fully protected against challenge [12].

In healthy human adults the addition of aluminum salt adjuvants to H5 vaccines have failed to consistently increase the immunogenicity of split virion vaccines [38]. The highest dose of vaccine with aluminum hydroxide adjuvant elicited the highest antibody titers but there were no consistent differences between adjuvanted and nonadjuvanted vaccinees overall [40]. Young adults had a greater response to an adjuvanted A/VN/1203 vaccine only at one dose in a dose ranging study [41]and two other studies found no adjuvant effect at any dose [42], [43]. Children developed ‘protective’ titers of clade-specific and cross-clade neutralizing antibody after two 30 µg alum-adjuvanted doses of a split-virus clade 1 vaccine [17]. Among healthy human adults immunogenicity measured by serum H5N1 antibody alone may not reflect protective efficacy, as has been shown in ferrets.

The reason for the disparate effects of aluminum adjuvants with H5 split virion vaccines between ferrets and humans is not clear. A reason may be pre-existing influenza immunity in older humans negates the alum advantage seen in young immunologically naïve seronegative ferrets. One distinct difference is the high frequency of heterosubtypic immunity from previous exposure to seasonal influenza that can provide some protection against a pandemic strain [3], [13], [44], [45], [46]. This heterosubtypic immunity has been thought to be mediated largely by T-cells [47]. Broad-spectrum neutralization targeting conserved epitopes on the hemagglutinin molecule [45], [48], [49] and the neuraminidase enzyme [50] may provide additional protection. Heterologous protection between clades has been observed due to the use of an oil-in-water adjuvant [10]. This suggests that the vaccine and adjuvant used for these studies may provide heterosubtypic immunity.

Serologically negative ferrets can express heterosubtypic immunity among seasonal strains [51]. A study of an unadjuvanted whole virion clade 1 vaccine in ferrets found homologous and heterologous protection even after a single low dose but these ferrets had previous exposures to H3N1 influenza virus [16]. However, we and other investigators used ferrets seronegative for HI antibody to circulating H3, H1 and type B strains both prior to shipment and immediately prior to the priming dose of vaccination. In addition, the pre-vaccination titers to H5 challenge antigens were negative, indicating immunologically naïve animals, within the sensitivity of the screening assays.

We found that some ferrets vaccinated without adjuvant with a 3-week interval had no detectable serum antibody at challenge yet survived lethal challenge (**Figure 1** **,** **2**). Other investigators have documented protection against lethal challenge in vaccinated ferrets in the absence of detectable neutralizing serum antibody measured by the HAI and MN assays [2], [10], [11], [36], [52]. Multiple mechanisms provide protection in the absence of serum antibody. The relative importance of humoral and cellular immunity in protection remains complex and difficult to predict [53]. We speculate that the shorter challenge interval, 3-weeks, may have assessed protection through an activated immune response. In recipients of seasonal trivalent vaccine there is an inverse relationship between neutralizing antibody titers and interferon-gamma-producing cells [54]. Multiple assays will be needed to construct a complete picture of the protective immune repertoire in the ferret [4], [55].

We observed here that vaccination without adjuvant afforded some protection after a 3-week booster-challenge interval, but no protection after a 4-week interval. No ferrets had detectable antibody at the time of challenge. We speculate that decreasing activated lymphocytes between 3 and 4 weeks explain the difference in survival. Results from this study need to be verified in another head-to-head comparison of booster-challenge intervals.

Increasing the booster-challenge interval of adjuvanted vaccine from one month to six months resulted in continued protection from mortality, yet without relief from weight loss, activity change, cytopenias, and fever. These pilot data within these studies are a beginning into the investigation of using similar vaccines for long term protection in areas with continual human infections and not just as near term protection prophylactic.

We examined ferret morbidity and the extent of inflammatory disease in the lung and brain in vaccinated animals (**Figure 8** **, S1, S2, S3**). Most ferrets vaccinated without adjuvant yet surviving to day 14 post-challenge had evidence of moderate to severe inflammation in both the lung and brain, indicating that extensive infection had progressed in the individual animal before an immune response was able to prevent death. Nasal wash results at day 2 and 4 demonstrate the lack of viral suppression. Moreover, all animals lost weight, experienced high fever, became anorectic, and exhibited decreased activity. Even survivors exhibited marked weakness, inactivity, thrombocytopenia, and weight loss 6 to 10 days after challenge before improving. The effect of the unadjuvanted vaccine in moderating disease was apparent by day 5 or 6 pi, late in the progression of disease and consistent with the onset of T-cell mediated immunity [56]. In contrast, ferrets that were vaccinated with alum adjuvant uncommonly showed morbidity. Slightly decreased activity was observed in only six animals and the lack of cytopenias, fever or significant weight loss corroborated minimal morbidity.

Comparison of the striking differences in brain inflammation between the unadjuvanted and alum-adjuvanted groups (**Figure 8**) reveals insights into the mechanism of increased protection mediated by the alum adjuvant. Reduction in spread of culturable virus to the ferret brain had been noted previously in a study of alum-adjuvanted whole virus vaccine [16]. In spite of diffuse brain inflammation in the survivors of the unadjuvanted vaccine groups all brain tissue cultures and viral RNA assays were negative, consistent with relatively late reversal of viral replication in the brain. Survivors of the alum-adjuvanted vaccine groups also had negative cultures but had no or minimal levels of brain inflammation. One reason for the lack of inflammation could be the high levels of neutralizing antibodies one week prior to challenge of the alum-adjuvanted vaccine groups. Yet, rather than suppressing the systemic viral replication, we suggest that since brain invasion appears to occur through sensory epithelial in the trubinates into the olfactory bulb [57] local mucosal immunity may be more important in preventing brain invasion.

The route of brain invasion and where protection may be mediated is not yet clear. In mice infected intranasally with H5N1 virus, spread to the brain was strain-dependent [58] and viral antigen appeared to enter via the mesenteric and myenteric plexi of the enteric nervous system prior to appearing in brainstem nuclei [59]. Direct injection of virus into the olfactory bulbs resulted in productive infection of brainstem neurons [60]. Olfactory bulb infection occurs in ferrets inoculated intranasally with high- and low- pathogenic H5N1 strains [30]. Ferrets infected with 1 to 7 log_10_ PFU of A/Vietnam/1203/04 and A/Hong Kong/483/97 strains had positive viral cultures in olfactory bulbs 24 h after intranasal inoculation and prior to appearance of virus in spleens and brains [31] (unpublished data) , and has been demonstrated by Shinya and colleagues [57]. In the turbinate tissue at 24 h pi viral antigen localized primarily to the sensory epithelial cells interfacing with neurons penetrating the cribiform plate to synapse in the olfactory bulbs [31] (S Kunder et al, unpublished observations). If brain invasion occurs within 24 h of viral challenge, alum-adjuvanted vaccine may stimulate mucosal immunity in the nasal turbinates to prevent early invasion. Unadjuvanted vaccine may fail to stimulate sufficient mucosal protection to prevent brain invasion, but in surviving ferrets residual activated systemic immunity arrested viral replication in the brain later in the course of disease.

In summary, the addition of alum adjuvant to a split-virion H5N1 vaccine resulted in marked improvement in mortality and morbidity. Further comparative studies of non-toxic adjuvants is urgently needed with particular attention to the correlation of protection to all humoral, cellular, and mucosal immune components of the host. Such strategies to overcome the reduced immunogenicity of split-virion avian influenza vaccines needs continued evaluation in animal models as well as safety and immunogenicity in humans.

## Supporting Information

Figure S1 **Representative pulmonary histopathology from ferrets (all panels 400× original magnification).** **A**) Normal lung for comparison (mild congestion of alveolar capillaries is present). **B**) Saline recipient animal at 6 pi with marked fibrinonecrotic pneumonia and loss of normal alveolar airspaces. Note abundant mixed inflammatory cells within septae and airspaces; pink, granular to fibrillar fibrin (f); hemorrhage; and karyorrhectic debris (small arrowheads). **C**) Unadjuvanted vaccine recipient at day 5 pi with similar fibrinonecrotic pneumonia and loss of normal alveolar airspaces. Note abundant inflammatory cells within septae and airspaces; pink, granular to fibrillar fibrin (f); hemorrhage; and karyorrhectic debris (small arrowheads). **D1**) Adjuvanted vaccine recipient at day 14 pi with perivascular mononuclear inflammation (large arrowhead) with abundant plasma cells as well as some septal infiltrates were often present in animals recovering from infection and examined at 2 weeks.
(TIF)

Figure S2 **Representative histopathology of olfactory bulb of brain of ferrets (all panels 400× original magnification).**Changes within olfactory bulbs were similar to those occurring in the remaining brain, though severity of the changes tended to diminish from rostral to caudal brain. **A**) Normal olfactory bulb for comparison. **B**) Saline recipient at day 6 pi with lymphohistiocytic and necrotizing meningoencephalitis. Note general hypercellularity due to inflammatory infiltrates and rarified areas due to spongiosus/edema (s). Eosinophilic “dead” neurons (arrowheads) are present along with abundant karyorrhectic debris (arrows). **C**) Unadjuvanted vaccine recipient at day 6 pi with similar changes to panel B. Note abundant inflammatory cells, eosinophilic “dead” neurons (arrowheads), spongiosus/edema (s), and abundant karyorrhectic debris (arrow). **D**) Adjuvanted vaccine recipient at day 14 pi with focal malacia/cavitating lesion within otherwise unremarkable olfactory bulb. The contralateral bulb was within normal limits and animal was clinically unremarkable. Moderate inflammatory/microglial cells remain within the area of tissue loss, including gitter cells (phagocytic cells containing lipid material from degenerating nervous tissue). Unadjuvanted vaccinees surviving to 14 days typically had similar, though generally larger and/or more abundant foci.
(TIF)

Figure S3 **Histopathology grading of lung lesions in survivors and non survivors.** “Lung –Acute” represents sections displaying active, fibrinonecrotic inflammation. “Lung –Subacute” represents sections found to have had evidence of prior or resolving inflammation. Tissues from moribund animals are denoted with an “M”, those from survivors (day 14 pi) are denoted with an “S”; the number after the “M” or “S” indicated the number of ferrets in that group. The Y-axis is the percent within each group (M or S) that was scored to each inflammation severity/grade. Grade 4 represents the most inflammation, whereas Grade 1 represents the least inflammation. Grade 0 is clear and represents that no inflammation was found. Each vaccination dose is labeled A–H.
(TIF)
