# Supplementary figures and images for: Enhanced Immunogenicity, Mortality Protection, and Reduced Viral Brain Invasion by Alum Adjuvant with an H5N1 Split-Virion Vaccine in the Ferret
Source: PLoS One. 2011 Jun 7;6(6):e20641. doi: 10.1371/journal.pone.0020641 (PMC3110201; doi:10.1371/journal.pone.0020641)

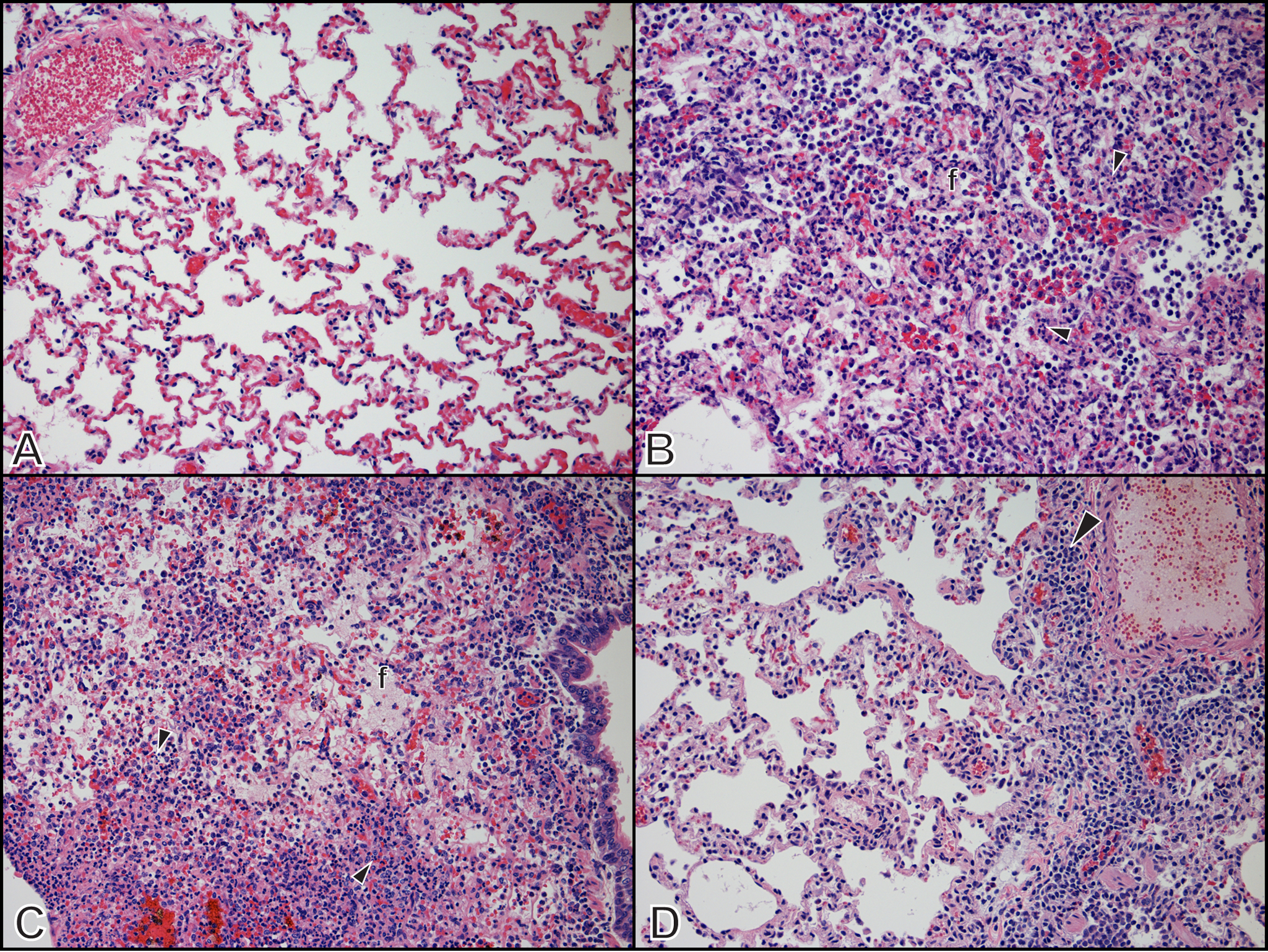

Supplement: Figure S1 — Representative pulmonary histopathology from ferrets (all panels 400× original magnification). A) Normal lung for comparison (mild congestion of alveolar capillaries is present). B) Saline recipient animal at 6 pi with marked fibrinonecrotic pneumonia and loss of normal alveolar airspaces. Note abundant mixed inflammatory cells within septae and airspaces; pink, granular to fibrillar fibrin (f); hemorrhage; and karyorrhectic debris (small arrowheads). C) Unadjuvanted vaccine recipient at day 5 pi with similar fibrinonecrotic pneumonia and loss of normal alveolar airspaces. Note abundant inflammatory cells within septae and airspaces; pink, granular to fibrillar fibrin (f); hemorrhage; and karyorrhectic debris (small arrowheads). D1) Adjuvanted vaccine recipient at day 14 pi with perivascular mononuclear inflammation (large arrowhead) with abundant plasma cells as well as some septal infiltrates were often present in animals recovering from infection and examined at 2 weeks. (TIF) [file pone.0020641.s001.tif]

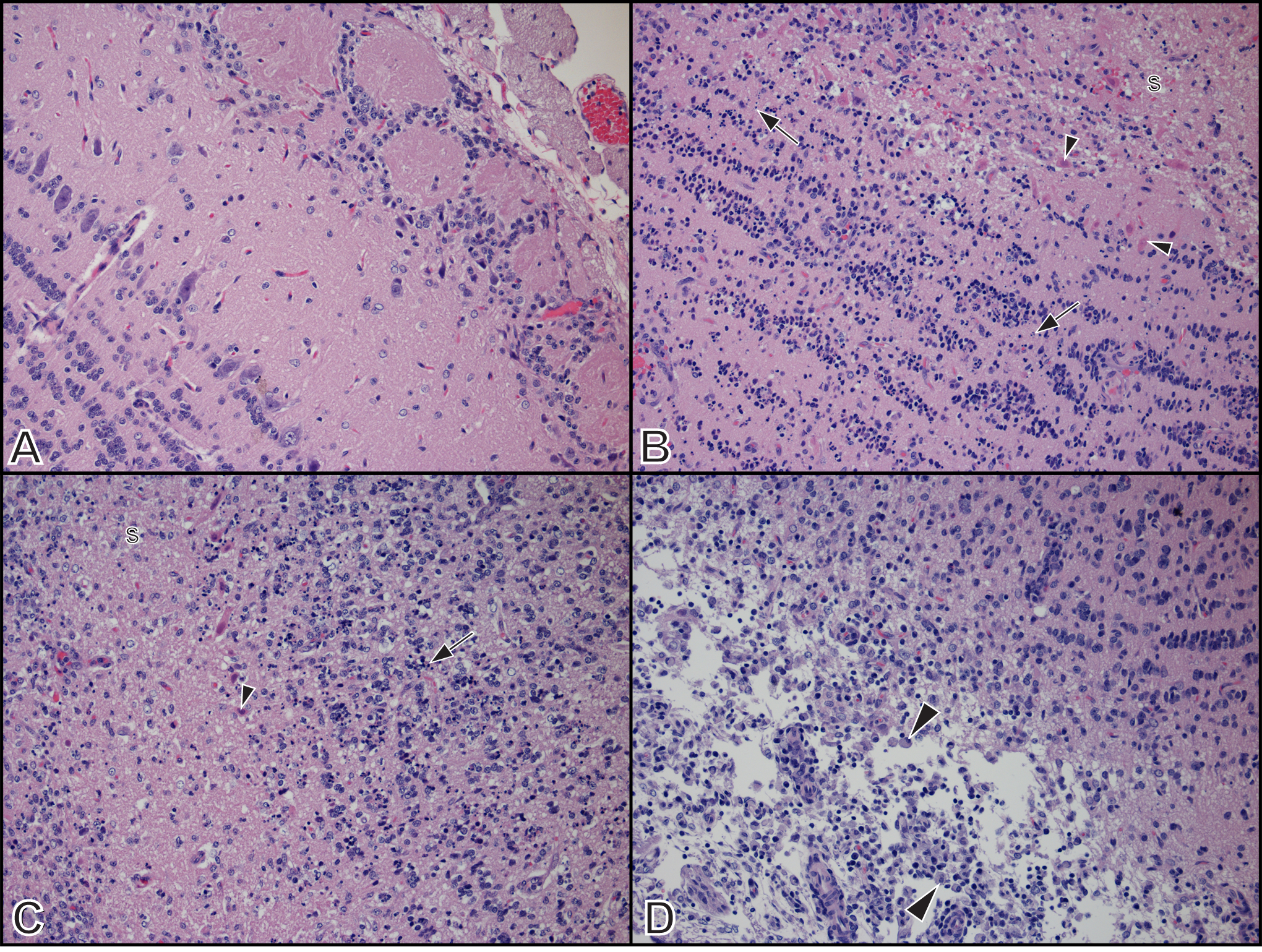

Supplement: Figure S2 — Representative histopathology of olfactory bulb of brain of ferrets (all panels 400× original magnification).Changes within olfactory bulbs were similar to those occurring in the remaining brain, though severity of the changes tended to diminish from rostral to caudal brain. A) Normal olfactory bulb for comparison. B) Saline recipient at day 6 pi with lymphohistiocytic and necrotizing meningoencephalitis. Note general hypercellularity due to inflammatory infiltrates and rarified areas due to spongiosus/edema (s). Eosinophilic “dead” neurons (arrowheads) are present along with abundant karyorrhectic debris (arrows). C) Unadjuvanted vaccine recipient at day 6 pi with similar changes to panel B. Note abundant inflammatory cells, eosinophilic “dead” neurons (arrowheads), spongiosus/edema (s), and abundant karyorrhectic debris (arrow). D) Adjuvanted vaccine recipient at day 14 pi with focal malacia/cavitating lesion within otherwise unremarkable olfactory bulb. The contralateral bulb was within normal limits and animal was clinically unremarkable. Moderate inflammatory/microglial cells remain within the area of tissue loss, including gitter cells (phagocytic cells containing lipid material from degenerating nervous tissue). Unadjuvanted vaccinees surviving to 14 days typically had similar, though generally larger and/or more abundant foci. (TIF) [file pone.0020641.s002.tif]

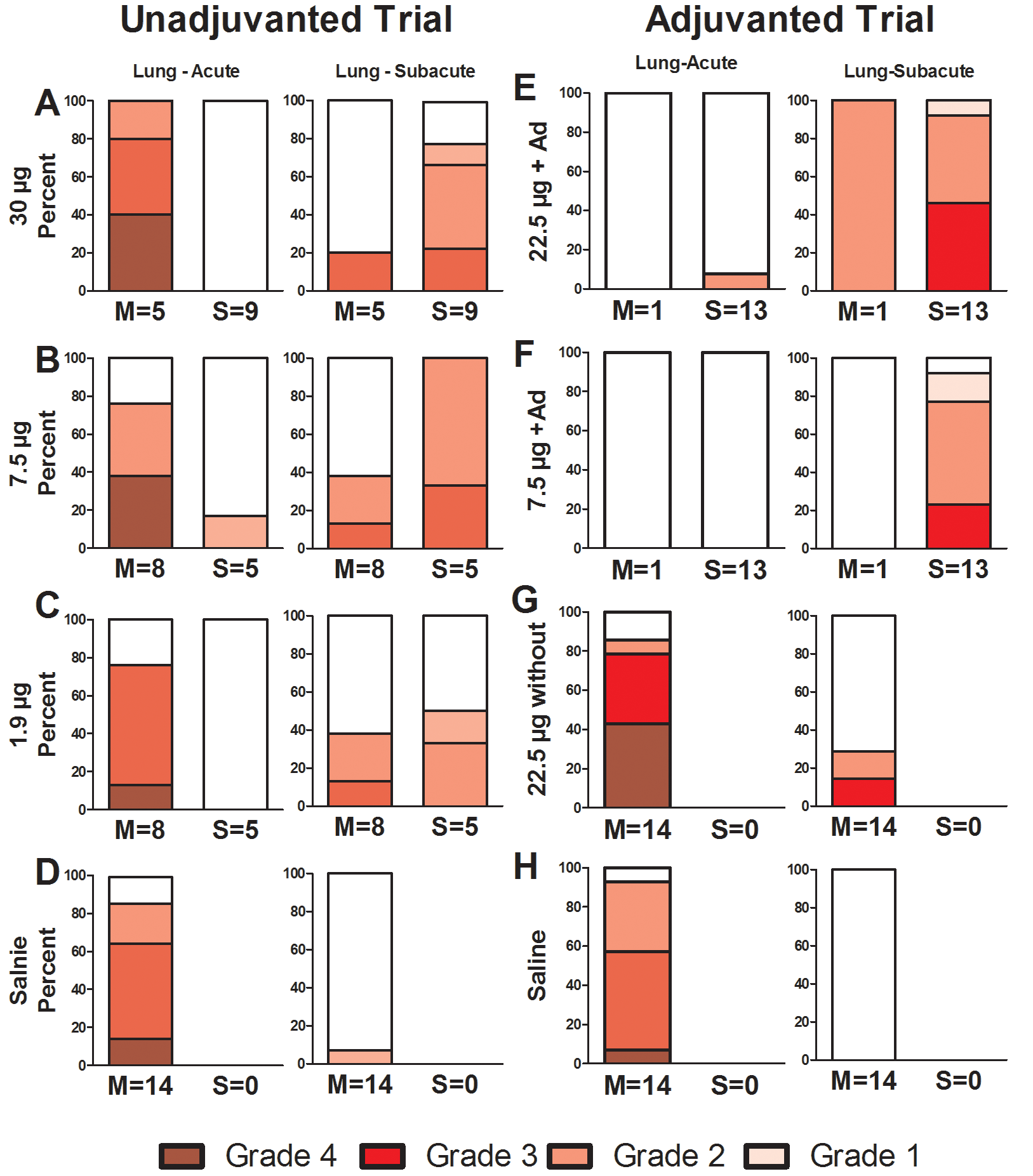

Supplement: Figure S3 — Histopathology grading of lung lesions in survivors and non survivors. “Lung –Acute” represents sections displaying active, fibrinonecrotic inflammation. “Lung –Subacute” represents sections found to have had evidence of prior or resolving inflammation. Tissues from moribund animals are denoted with an “M”, those from survivors (day 14 pi) are denoted with an “S”; the number after the “M” or “S” indicated the number of ferrets in that group. The Y-axis is the percent within each group (M or S) that was scored to each inflammation severity/grade. Grade 4 represents the most inflammation, whereas Grade 1 represents the least inflammation. Grade 0 is clear and represents that no inflammation was found. Each vaccination dose is labeled A–H. (TIF) [file pone.0020641.s003.tif]
